# Detection and size measurements of kidney stones on virtual non-contrast reconstructions derived from dual-layer computed tomography in an ex vivo phantom setup

**DOI:** 10.1007/s00330-022-09261-w

**Published:** 2022-11-23

**Authors:** R. P. Reimer, H. Zaytoun, K. Klein, K. Sonnabend, S. Lennartz, D. Zopfs, A. Heidenreich, D. Maintz, N. Große Hokamp

**Affiliations:** 1 Department of Diagnostic and Interventional Radiology, Faculty of Medicine and University Hospital Cologne, University of Cologne, Kerpener Str. 62, 50937 Köln, Cologne, Germany; 2 Philips GmbH Market DACH, Hamburg, Germany; 3 Department of Urology, Faculty of Medicine and University Hospital Cologne, University of Cologne, Cologne, Germany

**Keywords:** Contrast media, Tomography, spiral computed, Kidney calculi, Radiation dosage

## Abstract

**Objectives:**

To systematically investigate the usability of virtual non-contrast reconstructions (VNC) derived from dual-layer CT (DLCT) for detection and size measurements of kidney stones with regards to different degrees of surrounding iodine-induced attenuation and radiation dose.

**Methods:**

Ninety-two kidney stones of varying size (3–14 mm) and composition were placed in a phantom filled with different contrast media/water mixtures exhibiting specific iodine-induced attenuation (0–1500 HU). DLCT-scans were acquired using CTDI_vol_ of 2 mGy and 10 mGy. Conventional images (CI) and VNC_0H-1500HU_ were reconstructed. Reference stone size was determined using a digital caliper (Man-M). Visibility and stone size were assessed. Statistical analysis was performed using the McNemar test, Wilcoxon test, and the coefficient of determination.

**Results:**

All stones were visible on CI_0HU_ and VNC_200HU_. Starting at VNC_400 HU_, the detection rate decreased with increasing HU and was significantly lower as compared to CI_0HU_ on VNC_≥ 600HU_ (100.0 vs. 94.0%, *p* < 0.05). The overall detection rate was higher using 10 mGy as compared to 2 mGy protocol (87.9 vs. 81.8%; *p* < 0.001). Stone size was significantly overestimated on all VNC compared to Man-M (7.0 ± 3.5 vs. 6.6 ± 2.8 mm, *p* < 0.001). Again, the 10 mGy protocol tended to show a better correlation with Man-M as compared to 2 mGy protocol (*R*^2^ = 0.39–0.68 vs. *R*^2^ = 0.31–0.57).

**Conclusions:**

Detection and size measurements of kidney stones surrounded by contrast media on VNC are feasible. The detection rate of kidney stones decreases with increasing iodine-induced attenuation and with decreasing radiation dose as well as stone size, while remaining comparable to CI_0HU_ on VNC _≤ 400 HU_.

**Key Points:**

*• The detection rate of kidney stones on VNC depends on the surrounding iodine-induced attenuation, the used radiation dose, and the stone size.*

*• The detection rate of kidney stones on VNC decreases with greater iodine-induced attenuation and with lower radiation dose, particularly in small stones.*

*• The visibility of kidney stones on VNC*_≤ *400 HU*_ *remains comparable to true-non-contrast scans even when using a low-dose technique.*

**Supplementary Information:**

The online version contains supplementary material available at 10.1007/s00330-022-09261-w.

## Introduction

Hematuria and urolithiasis rank among the most common urologic diagnoses, with urolithiasis being one of the leading causes of hematuria [1, 2]. While the prevalence of hematuria has been reported between 2.4 and 31.1%, the lifetime prevalence of urolithiasis is approximately 15% in developed countries [2, 3]. In patients with suspected urolithiasis, non-contrast computed tomography (CT) and in patients with hematuria and a high risk for genitourinary malignancy, multiphasic CT is recommended by international guidelines authored by the European Association of Urology (EAU) and the American Urological Association, respectively [3, 4]. Due to the rising prevalence of urolithiasis and a high rate of recurrence of up to 50%, limiting radiation exposure in these and other urologic patients is a major concern [4, 5].

In recent decades, technical advances allowed for extensive investigations regarding the reduction of radiation dose in computed tomography. This leads to the implementation of low-dose scanning protocols for patients with suspected urolithiasis in the international guideline by the EAU [4, 6–8]. On the contrary, the radiation dose of multiphasic CT in patients with hematuria including a non-contrast, parenchymal and excretory phase still exhibits a high radiation dose. Here, virtual non-contrast reconstructions (VNC) of excretory phase derived from dual-energy CT systems showed promising results in urinary stone detection to replace true non-contrast scans [9, 10]. More recently, split-bolus protocols have been described to further reduce radiation dose by combining the parenchymal and excretory phase of the multiphasic hematuria CT protocol. Hence, when applied to dual-energy CT (DECT) systems, the non-contrast, parenchymal and excretory phase may be obtained within a single scan yielding a radiation dose reduction of up 40 to 60% [11, 12]. So far, previous studies investigated the detection of kidney stones on VNC of the excretory phase using the different available emission-based DECT systems. To the best of our knowledge, there is no study available investigating the effect of different parameters using VNC of the dual-layer DECT system (DLCT). In particular, information on the performance of DLCT regarding detectability and size measurements of kidney stones is unavailable; however, both frequently determine treatment strategies in patients with kidney stones [1, 4, 12, 13].

Hence, the aim of this study was to investigate the effects of different degrees of surrounding iodine-induced attenuation and radiation dose on the detection rate and size measurements of kidney stones on VNC obtained from DLCT.

## Material and methods

This retrospective study was classified as non-human research by the local institutional review board. Kidney stones are routinely collected from surgical treatment procedures and chemical stone composition is routinely analyzed at the local laboratory using infrared spectroscopy. The reference standard of the stone size for subsequent analyses was obtained using a mean out of two measurements of the long-axis diameter with a vernier caliper (Man-M). For this study, 96 stones with a minimum long-axis diameter of 3 mm were initially included, of which 4 stones fractured during handling and were therefore excluded from the final analysis. Hence, this study comprised 92 kidney stones representing the clinically encountered variability: brushite (*n* = 11), cystine (*n* = 10), dahllite (*n* = 11), struvite (*n* = 7), uric acid (*n* = 12), weddellite (*n* = 12), whewellite (*n* = 20), and xanthine stones (*n* = 9) (Table 1).

**Table 1:** Manual measurements of the longest diameter regarding different kidney stone compositions of all stones and manual measurements of the subset for the comparison of detection rate between different stone compositions. Results are indicated as mean ± standard deviation (range)

Stone composition	N	Man-M (mm)	*N*, subset	Man-M, subset (mm)
Brushite	11	5.3 ± 1.9 (3.2–8.1)	5	6.4 ± 1.3 (5.0–7.9)
Cystine	10	7.9 ± 3.1 (3.3–12.5)	4	6.3 ± 2.0 (5.0–9.4)
Dahllite	11	4.7 ± 1.1 (3.0–6.7)	2	6.2 ± 0.6 (5.8–6.7)
Struvite	7	5.4 ± 2.0 (3.2–8.1)	5	6.3 ± 1.6 (5.0–8.1)
Uric acid	12	6.5 ± 2.9 (3.2–13.4)	5	6.3 ± 0.7 (5.3–7.1)
Weddellite	12	8.4 ± 3.3 (4.0–14.0)	3	6.4 ± 1.8 (5.2–8.5)
Whewellite	20	5.6 ± 2.4 (3.2–12.1)	7	6.3 ± 1.0 (5.5–8.0)
Xanthine	9	9.3 ± 1.8 (6.3–11.6)	1	6.3

N, number; *Man-M*, manual measurements

### Phantom design

**Fig. 1:**
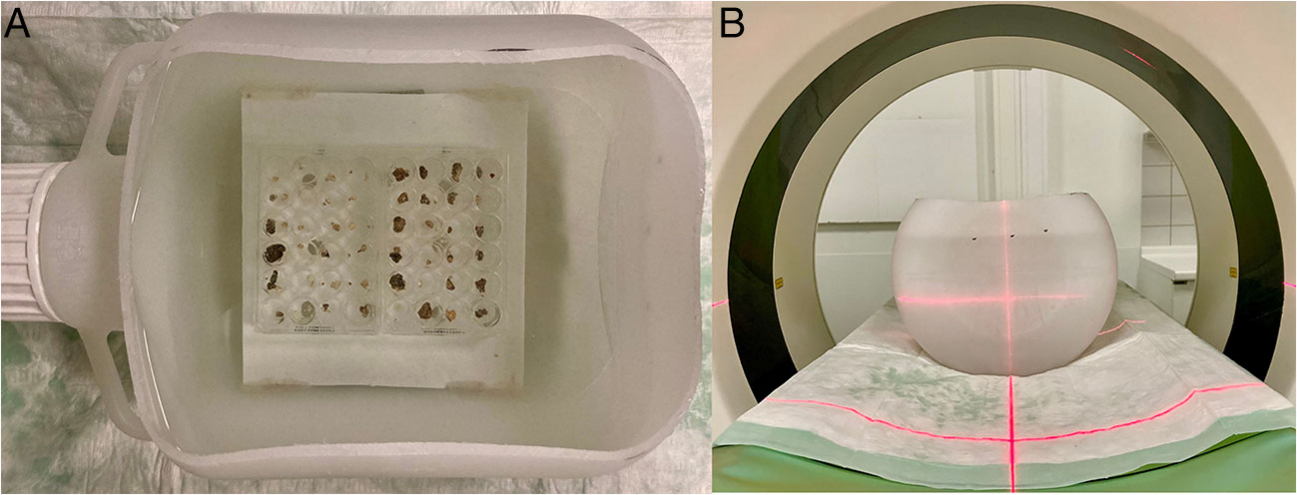
Water phantom with kidney stones placed on gelatine in well plates filled with different contrast media/saline mixtures yielding specific iodine-induced attenuation of 0–1500 HU (**A**). Subsequently, CT scans were performed using a CTDI_vol_ of 2 and 10 mGy (**B**). This was repeated for each scan with a different iodine-induced attenuation

For all examinations, the stones were placed on a layer of gelatine (Oetker) exhibiting water-equivalent HU within polystyrene 24-well plates (surface area 1.9 cm^2^; Corning Inc), to ensure a sufficient distance to the base plate for accurate stone size assessment using semi-automatic segmentation. The well plates were then filled with increasing amounts of iohexol (Accupaque, GE Healthcare) in water yielding an iodine-induced attenuation of 0 HU, 200 HU, 400 HU, 600 HU, 800 HU, 1000 HU, and 1500 HU. Corresponding contrast media/water ratios were calculated based on a linear formula derived from a prior performed dilution series. The well plates were placed on a platform in a plastic box filled with water (width x height: 280 x 200 mm), which was positioned along the z-axis on the CT table and centrally within the gantry (Fig. 1). The procedure was repeated for each scan with a different iodine-induced attenuation. In between each scan with increasing iodine-induced attenuation, kidney stones were washed in saline to minimize possible absorption and accumulation of contrast media on the surface of the stones. Prior to each scan, a test scan of the different contrast media/water mixtures was acquired to ensure accurate iodine-induced attenuation and mixtures were adjusted if necessary. Attenuation values were quantified by placing two circular regions of interest within the mixtures. Here, attenuation values ± 10 HU of the desired iodine-induced attenuation were considered sufficient (ESM 1).

### Scanning parameters and image acquisition

All scans were performed on a clinical DLCT scanner (IQon, Philips Healthcare). Tube current time products were set at 22 and 111 mAs resulting in a volumetric computed tomography dose index (CTDI_vol_) of 2 and 10 mGy, respectively. Other relevant scan parameters were as follows: tube voltage = 120 kVp, collimation = 64 x 0.625 mm, matrix = 512 x 512, rotation time 0.5 s, and pitch 0.578. Conventional images (CI) were reconstructed for scans with water (CI_0HU_) using a hybrid-iterative reconstruction algorithm (iDose^4^, kernel B, level 3, Philips Healthcare). VNC were reconstructed for scans with iodine-induced attenuation of 200–1500 HU (VNC_200-1500HU_) using a dedicated spectral reconstruction algorithm (Spectral, kernel B, level 3; Philips Healthcare). Images were reconstructed in axial reorientation using a slice thickness and section increment of 0.67 mm, respectively.

### Image analysis

Data sets were saved in DICOM format. Image analysis was performed on CI_0HU_ and VNC_200-1500HU_ using a soft-tissue window setting (width = 360 HU, level = 60 HU). First, two readers with two and five years of experience in abdominal imaging assessed the visibility of kidney stones in a consensus reading using a clinical DICOM viewer (Impax EE R20, Dedalus Group). Second, all visible stones were semi-automatically segmented by the first reader using an attenuation threshold-based algorithm implemented in an open-source DICOM image viewer (Horos v3.0, licensed under GNU Lesser General Public License). In a chosen subset of 24 stones comprising three of each available stone composition, the second reader repeated the segmentation to assess interrater reliability. The lower cut-off value was defined as 50 HU and was adjusted if needed to prevent the inclusion of any surrounding tissue, respectively. The upper cut-off value was set to maximum (i.e., 3072 HU). To assess the maximum diameter of each 3-dimensional volume of interest of every visible stone, an in-house developed script was employed (MATLAB 2019a, The MathWorks, Inc.) [14]. The two readers were free to adjust window settings when determining visibility and segmentation correctness

### Statistical analysis

Statistical analyses were performed using Microsoft Excel (Version 16.57, Microsoft Corporation), JMP Software (Version 16.1.0, SAS Institute), SPSS Software Statistics (Version 28, IBM), and R Studio (Version 2021.09.01, RStudio Inc.). A *p* value < 0.05 was considered significant. To compare the detection rate between CI_0HU_ and VNC_200-1500HU_ as well as CTDI_vol_ of 2 and 10 mGy, the McNemar test was applied. To compare the detection rate between the different stone compositions and stone sizes, fisher’s exact test was applied. To compare detection rates between different stone compositions, a subset of 32 stones with comparable manually measured stone sizes were chosen (Table 1).

Stone size measurements were compared using Wilcoxon signed rank test. The correlation between CT measurements of the longest diameters and Man-M was determined using the coefficient of determination. Interrater reliability was determined by means of intra-class correlation estimates (intra-class correlation coefficient (ICC)) based on a single rater, consistency, 2-way mixed-effects model for the CT-based size measurements [15]. It was evaluated as described earlier: excellent (ICC > 0.8), good (ICC > 0.6), moderate (ICC > 0.4), and poor agreement (ICC < 0.4) [16].

## Results

### Kidney stone detection

**Fig. 2:**
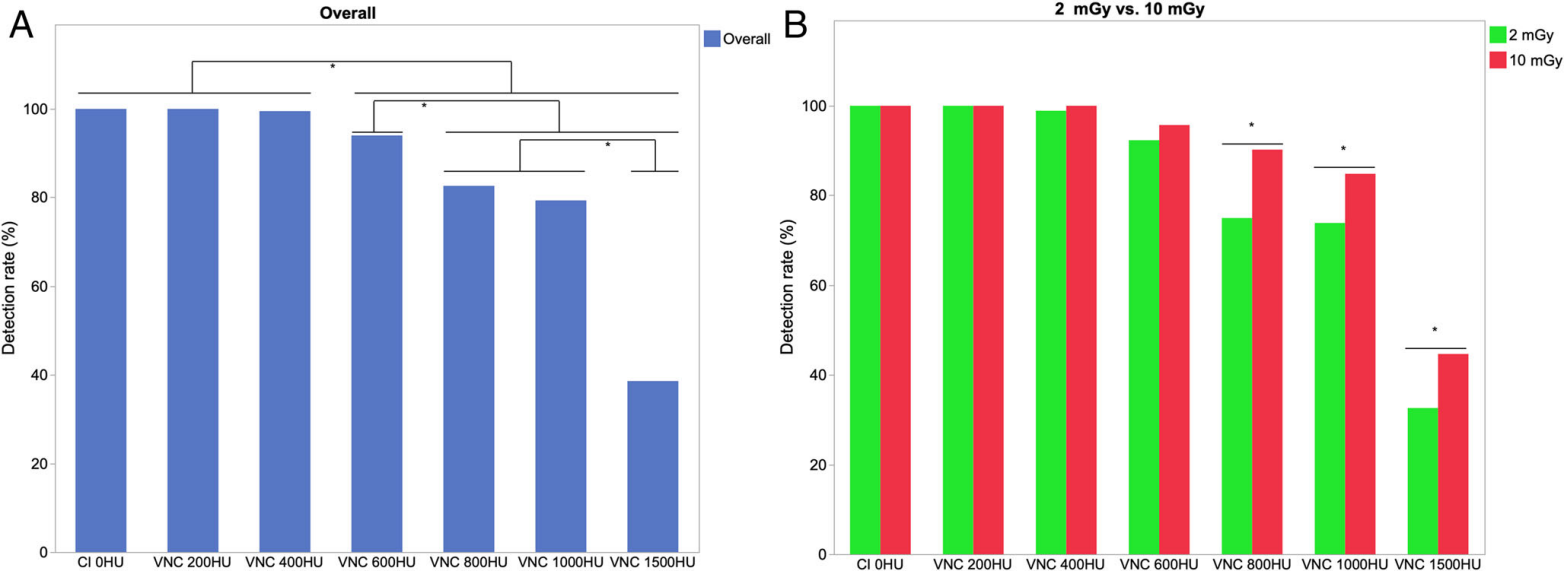
Bar diagrams illustrating the detection rate of kidney stones on conventional images (CI) surrounded by water and on virtual non-contrast reconstructions (VNC) surrounded by increasing iodine-induced attenuation from 200 to 1500 HU (**A**, **B**). The overall detection rate decreased continuously starting at VNC_400 HU_ (**A**). Regarding similar iodine-induced attenuation, the detection rate was higher in scans using the high-dose protocol (10 mGy) as compared to the low-dose protocol (2 mGy) on VNC_≥__400 HU_ (**B**)

**Fig. 3:**
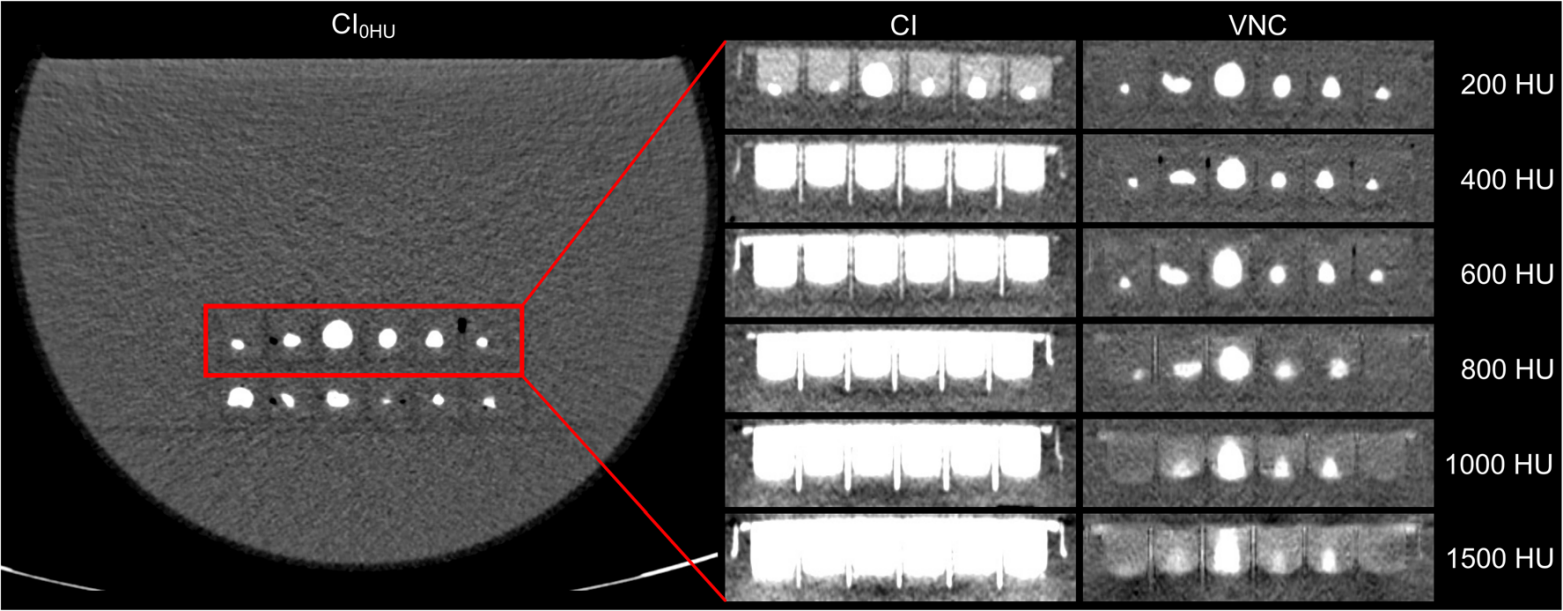
Dual-layer CT phantom scans of the same kidney stones using 10 mGy with different contrast media/saline mixtures exhibiting specific iodine-induced attenuation from 0 HU to 1500 HU with corresponding conventional images (CI) and virtual non-contrast reconstructions (VNC). Kidney stones can be well depicted on VNC _≤ 600HU_, whereas stone size proportions are not comparable between the different scans of these image examples due to different stone positions within each well plate

All stones were detected on CI_0HU_ and VNC_200HU_. Starting at VNC_400 HU_, the detection rate decreased on VNC with increasing iodine-induced attenuation and was significantly lower on VNC _≥ 600HU_ as compared to CI_0HU_ and VNC _≤ 400 HU_ (*p* < 0.001). Simultaneously, the detection rate on VNC_600HU_ was significantly higher as compared to VNC_≥ 800HU_ as well as significantly higher on VNC_800HU_ and VNC_1000HU_ as compared to VNC_1500HU_ (*p* < 0.001, respectively) (Table 2 and Figs. 2A and 3).

**Table 2:** Detection rate of kidney stones in the different contrast media/saline mixtures exhibiting specific iodine-induced attenuation using a low- and a high-dose protocol

Reconstruction	Detection rate, overall (%)	Detection rate, 2 mGy (%)	Detection rate, 10 mGy (%)	*p* value, 2 vs. 10 mGy
CI_0HU_	100	100	100	-
VNC_200HU_	100	100	100	-
VNC_400 HU_	99.5	98.9	100	1
VNC_600HU_	94.0	92.3	95.7	0.250
VNC_800HU_	82.6	75.0	90.2	< 0.001
VNC_1000HU_	79.3	73.9	84.8	< 0.001
VNC_1500HU_	38.6	32.6	44.6	0.003

*CI*, conventional images; *HU*, Hounsfield unit; *VNC*, virtual non-contrast reconstructions

### Influence of radiation dose on detection rate

The overall detection rate of kidney stones was significantly higher in the high-dose protocol as compared to the low-dose protocol (87.9 vs. 81.8%; *p* < 0.001). Regarding similar iodine-induced attenuation, the detection rate was higher in scans using the high-dose protocol as compared to the low-dose protocol on VNC_≥400 HU_ reaching a statistical significance at VNC_≥ 800HU_ (VNC_800HU_: 90.2% vs. 75.0%, *p* < 0.001) (Table 2 and Fig. 2B). The detection rate remained comparable to CI_0HU_ on VNC_≤ 400 HU_ when using the low-dose (2 mGy) and on VNC_≤ 600HU_ when using the high-dose protocol (10 mGy) (*p* < 0.05).

### Influence of stone composition on detection rate

The detection rate was highest for dahllite stones (89.3%), followed by weddellite (88.1%), whewellite (85.7%), xanthine (85.7%), brushite (84.3%), uric acid (78.6%), cystine (76.8%), and struvite stones (75.7%) without any statistical differences between different stone compositions (*p* > 0.05).

### Kidney stone size measurements

Regarding CT-based size measurements, mean ICC was 0.923 (0.907–0.936), indicating excellent interrater reliability. Across all stones, the manually measured size was 6.7 ± 2.8 mm ranging from 3.0 to 14.0 mm, while CT-based measurements were higher when averaging all reconstructions (7.1 ± 3.5 mm; *p* < 0.001). The overall correlation between Man-M and CT-based measurements was good (*p* < 0.001, *R*^2^ = 0.47). Regarding specific iodine-induced attenuation, CT-based measurements showed a good correlation with Man-M (*R*^2^ = 0.40–0.58; *p* < 0.001), while measurements using the high-dose protocol showed a better correlation than using the low-dose protocol (*R*^2^ = 0.39–0.68 vs. *R*^*2*^ = 0.31–0.57; all *p* < 0.001) (Table 3). Yet, CT-based measurements of stone size did not significantly differ between scans with 2 mGy and 10 mGy when averaging all reconstructions (7.2 ± 3.6 vs. 7.0 ± 3.3 mm; *p* > 0.05).

**Table 3:** Stone measurements in different contrast media/saline mixtures exhibiting specific iodine-induced attenuation using a low- and a high-dose protocol

Reconstruction	Man-M (mm)	CT-based, overall (mm) [*R*^2^]	CT-based, 2 mGy (mm) [*R*^2^]	CT-based, 10 mGy (mm) [*R*^2^]
CI_0HU_	6.7 ± 2.8 (3.0–14.0)	7.8 ± 3.1 (3.0–16.0) [0.52]	8.0 ± 3.1 (3.6–16.3) [0.50]	7.5 ± 3.0 (3.1–16.2) [0.53]
VNC_200HU_		7.1 ± 3.3 (2.3–16.3) [0.56]	7.1 ± 3.4 (2.3–16.1) [0.56]	7.1 ± 3.2 (2.7–16.3) [0.56]
VNC_400 HU_		7.2 ± 3.5 (1.8–16.6) [0.58]	7.3 ± 3.5 (1.8–15.9) [0.57]	7.2 ± 3.4 (2.0–16.6) [0.58]
VNC_600HU_		7.5 ± 3.8 (1.9–17.6) [0.52]	7.6 ± 4.1 (1.9–17.4) [0.49]	7.3 ± 3.5 (2.5–17.6) [0.57]
VNC_800HU_		5.8 ± 3.4 (1.9–14.9) [0.38]	5.7 ± 3.4 (1.9–14.9) [0.38]	5.9 ± 3.3 (2.0–14.9) [0.39]
VNC_1000HU_		6.5 ± 3.9 (1.9–15.3) [0.40]	6.1 ± 3.7 (1.9–15.1) [0.31]	6.6 ± 3.2 (1.9–15.3) [0.51]
VNC_1500HU_		8.3 ± 3.5 (2.0–17.1) [0.51]	9.4 ± 3.3 (2.7–15.2) [0.32]	7.6 ± 3.4 (2.0–17.1) [0.68]

*Man-M*, manual measurements; *CT*, computed tomography; *CI*, conventional images; *HU*, Hounsfield units; *VNC*, virtual non-contrast reconstructions. Results are indicated as mean ± standard deviation (range) [Coefficient of determination]

### Influence of kidney stone size on detection rate

Considering a general cut-off for insignificant stones of ≤ 5 mm described by current guidelines [4, 17], 32 kidney stones (34.8 %) were ≤ 5 mm, while 60 kidney stones (65.2 %) were > 5 mm (indicated by Man-M, respectively). The overall detection rate of kidney stones ≤ 5 mm was significantly lower as compared to stones > 5 mm (80.6 % vs. 87.1 %; *p* < 0.01) when averaging all reconstructions. With increasing iodine-induced attenuation, detection rates in low-dose protocols dropped sooner as compared to high-dose protocols (e.g., VNC_1000HU_ 68.8 % vs 85.0 %, *p* < 0.01) (Table 4).

**Table 4:** Detection rate of kidney stones in the different contrast media/saline mixtures exhibiting specific iodine-induced attenuation regarding kidney stone size

Reconstruction	Detection rate, overall (%)	Detection rate, ≤ 5 mm (%)	Detection rate, > 5 mm (%)	*p* value, ≤ vs. > 5 mm
CI_0HU_	100	100	100	-
VNC_200HU_	100	100	100	-
VNC_400 HU_	99.5	100	99.2	1
VNC_600HU_	94.0	92.2	95.0	0.518
VNC_800HU_	82.6	78.1	85.0	0.307
VNC_1000HU_	79.3	68.8	85.0	0.013
VNC_1500HU_	38.6	25.0	45.8	0.007

*CI*, conventional images; *HU*, Hounsfield unit; *VNC*, virtual non-contrast reconstructions

## Discussion

This study investigated the effects of different degrees of surrounding iodine-induced attenuation and radiation dose on the detection rate and size measurements of kidney stones on VNC obtained from DLCT. We found that the overall detection rate continuously decreased with increasing surrounding iodine-induced attenuation, while remaining comparable to true-non-contrast scans on VNC _≤ 400 HU_ even when using a low-dose technique. The detection rate of kidney stones was higher in the high-dose protocol as compared to the low-dose protocol and was comparable between both protocols on VNC _≤ 600HU_. Kidney stone size influenced the detection rate and was generally overestimated. Stones > 5 mm were better detected than stones ≤ 5 mm, while the detection rate remained comparable on VNC _≤ 800HU_.

The reported data demonstrate the beneficial value of VNC derived from dual-energy CT in the imaging of hematuria and urolithiasis. VNC has the potential to replace the true non-contrast scan in multiphasic CT protocols resulting in a reduction of radiation dose, while maintaining a high detection rate of kidney stones when taking the observed limitations into account. To the best of our knowledge, our study represents the first study investigating the detection of kidney stones on VNC derived from DLCT, while most published in vivo studies used rapid-switching rather than dual-source DECT [18]. A direct translation of the presented results to other DECT systems is limited due to different technical aspects, such as a reported variation in iodine quantification accuracy between the different DECT systems—which, in turn, closely relates with VNC accuracy [19, 20]. Yet it has been shown that iodine can be accurately quantified with all state-of-the-art DECT systems including DLCT [19–21]. Nonetheless, future studies are encouraged to perform a true head-to-head comparison.

Earlier studies already reported an insufficient subtraction of iodine and a decrease in the detection rate of kidney stones at an attenuation of 600–730 HU, which is in line with the results of this study [1, 22]. However, the luminal attenuation in the renal pelvis during the excretory phase may exceed these values possibly hampering kidney stone detection on VNC [10]. Hence, different split-bolus protocols have been investigated to limit the luminal attenuation in the renal pelvis, e.g., Chen et al were able to limit the attenuation of the renal pelvis to 457.2 ± 168.8 HU and reported a detection rate of 87.5 % on VNC when using a bolus of 200 mL saline, followed by 50 mL contrast media (300 mg iodine/mL) 6 minutes prior to the second bolus of 70 mL contrast media, followed by 24 mL saline and scanning delay of 60 seconds [23]. More recently, McCoombe et al published a systematic review and meta-analysis of in-vivo studies on the detection rate of kidney stones on VNC of excretory phase obtained from split-bolus DECT. The authors reported the highest pooled sensitivity of 92.2 % on VNC compared to true non-contrast scans in a subgroup analysis of studies that used oral hydration and < 2 mm slice thickness [18]. Future studies should take vital parameters such as heart rate and blood pressure as well as the glomerular filtration rate into account when investigating new optimization possibilities of split-bolus protocols as these might affect the excretion of contrast agent.

Other studies demonstrated that kidney stone size influences the detection rate on VNC [1, 9, 10, 24]. In line with these studies, we found a difference in size between visible and invisible stones as well as a positive correlation between stone size and detectability. Considering a general cut-off for insignificant stones of ≤ 5 mm described by current guidelines [4, 17], our results showed a significantly better detectability for stones > 5 mm, which remained as high as 85 % on VNC_1000HU_. Yet, it has to be mentioned that stones smaller than 5 mm might also cause symptoms and might not pass spontaneously, although up to 95 % of kidney stones smaller than 4 mm pass spontaneously [4, 17, 25]. More particular detection of small stones may be diagnostic in the scenario of unclear hematuria (irrespective of the impact on treatment).

Furthermore, previous studies reported an underestimation of kidney stone size on VNC compared to true non-contrast images due to an over-subtraction of small stones or of the peripheral stone along with the iodine signal [1, 9, 10]. Contrary, CT-based stone size in this study was generally overestimated compared to Man-M, which is e.g. discrepant to an earlier ex-vivo study of Lazar et al using a DSCT system, performing 2D-based size measurements on axial reconstructions and including smaller stones with a diameter of 2.4 ± 1.1 mm in which the over-subtraction may have a higher influence on size measurements [1]. A possible explanation for this behaviour might lie in the different scanner techniques and hence post-processing capabilities used between the studies.

Additionally, it has been shown that measurements taking the three-dimensional irregular structure of kidney stones by means of volumetric CT-based measurements or multiplanar reconstructions into account might be a better predictor of treatment outcome and showed closer agreement with true stone size [26–28].

Besides, the detectability and size assessment of small kidney stones using low-dose protocols in the non-contrast imaging of urolithiasis as recommended by current guidelines has been extensively investigated in previous studies. These studies demonstrated high diagnostic accuracy and accurate size assessment of small stones with a diameter of less than 3 mm as compared to high-dose protocols [7, 14, 29, 30]. Pertaining to the detection of kidney stones surrounded by iodine-induced attenuation on VNC, our study showed comparable detectability between the low- and high-dose protocol on VNC_≤__600HU_ as well as comparable size measurements. Hence, a further reduction of radiation dose in clinical studies of the imaging of hematuria seams conceivable, since previous in-vivo and ex vivo studies only investigated radiation doses as low as 6 and 10 mGy [1, 10].

In addition, non-contrast dual-energy CT showed better differentiation of stone composition compared to polyenergetic CT, even when using a low-dose technique [31, 32]. So far, only Lazar et al analysed the effect of stone composition on the detection rate of kidney stones on VNC and reported the best detectability for cystine stones, followed by struvite, brushite, and whewellite stones [1]. On the contrary, we found higher detection rates for calcium (whewellite, weddellite, dahllite, brushite) and xanthine stones (> 80%) as compared to uric acid, cystine and struvite stones (< 80%) without reaching a statistical significance. Besides, DECT type-dependent differences, these discrepancies may also result from the inclusion of larger stones in our analysis.

In light of the presented data and discussed literature, VNC may reduce the radiation dose in the imaging of hematuria by replacing TNC if the surrounding attenuation in the renal pelvis is ≤ 400 HU irrespective of radiation dose. A possible clinical approach in unknown hematuria consists of a single acquisition split-bolus protocol including the interpretation of VNC images and, in case of no identifiable cause of hematuria and the attenuation in the renal pelvis exceeding 400 HU, an additional low-dose TNC acquisition can be considered for increased detection of small stones, which might have clinical relevance in this specific contrast as a cause of hematuria.

Apart from the limited number of stones included per stone composition, there are several limitations to this study. First, this is an ex vivo study. However, the highly standardized phantom setup allowed us to perform a thorough investigation of the different parameters, which would not be possible in an in-vivo setting. Second, we adopted the radiation dose protocols from previous studies and from our institutional settings [8, 26, 31]. However, the image quality obtained in our ex vivo setup may not translate into in-vivo applications. While the used phantom diameter of 280 mm is close to the diameter of the general body phantom (320 mm) used for CT dosimetry, the homogeneous attenuation of water does not represent in vivo conditions and possibly results in an underestimation of radiation dose as compared to the in-vivo setting [33]. Furthermore, it is known that the detection of kidney stones on TNC is hampered in patients with high body mass indices due to a decrease in image quality, which is yet to be shown for VNC [34]. Last, the ex vivo conditions resulted in a bias as to in which position a stone would be expected and will therefore likely overestimate detection rates as compared to a true patient study. Studies addressing this in-vivo detectability of renal stones are encouraged in order to adapt our findings into clinical workflow.

To conclude, detection and size measurements of kidney stones surrounded by contrast media on VNC are feasible, which may reduce radiation dose by replacing true non-contrast scans. The detection rate of kidney stones depends on the surrounding iodine-induced attenuation, the used radiation dose, and kidney stone size. In particular, stones are expected to be detectable in VNC if surrounding attenuation is ≤ 400 HU irrespective of radiation dose.

## Supplementary information

ESM 1 (DOCX 22 kb)

## Abbreviations

CI: Conventional reconstructions
CT: Computed tomography
CTDI_vol_: Volumetric computed tomography dose index
DECT: Dual-energy computed tomography
DLCT: Dual-layer computed tomography
Man-M: Manual measurements
VNC: Virtual non-contrast reconstructions

## Declarations

### Guarantor

The scientific guarantor of this study is PD Dr. med. Nils Große Hokamp.

### Statistics and biometry

RPR, SL, and NGH have significant statistical expertise.

### Methodology

• retrospective

• experimental

• performed at one institution
