# Supplementary material for: Detection and size measurements of kidney stones on virtual non-contrast reconstructions derived from dual-layer computed tomography in an ex vivo phantom setup
Source: Eur Radiol. 2022 Nov 23;33(4):2995–3003. doi: 10.1007/s00330-022-09261-w (PMC10017605; doi:10.1007/s00330-022-09261-w)
Supplement: Supplementary file 1 — (DOCX 22 kb) [file 330_2022_9261_MOESM1_ESM.docx]

ESM 1. Measured attenuation values of the different contrast media/water mixtures.

| Reconstruction | Attenuation value [HU] |
| --- | --- |
| CI_0HU_ | 0.4 ± 0.3 |
| VNC_200HU_ | 201.0 ± 1.4 |
| VNC_400HU_ | 407.0 ± 1.4 |
| VNC_600HU_ | 607.5 ± 3.5 |
| VNC_800HU_ | 807.5 ± 4.9 |
| VNC_1000HU_ | 999 ± 1.4 |
| VNC_1500HU_ | 1498.5 ± 2.1 |

HU, Hounsfield unit
